# The Prevalence of Vitamin D Deficiency in Patients Undergoing Total Knee Arthroplasty: A Propensity Score Matching Analysis

**DOI:** 10.1007/s11657-022-01097-7

**Published:** 2022-03-23

**Authors:** Young-Dae Jeon, Sung-Do Cho, Yoon-Seok Youm, Joon-Yeon Song, Kyung-Joo Lee, Ki-Bong Park

**Affiliations:** 1 Department of Orthopaedic Surgery, Ulsan University Hospital, University of Ulsan College of Medicine, 877 Bangeojinsunhwando-ro, Dong-gu, Ulsan, 44033 Republic of Korea; 2 Department of Orthopaedic Surgery, Dongcheondongkang Hospital, Ulsan, Republic of Korea; 3 Department of Orthopaedic Surgery, Dongguk University Ilsan Hospital, Goyang-si, Gyeonggi-do Republic of Korea

**Keywords:** 25-OH vitamin D, Knee, Osteoarthritis, Total knee arthroplasty, Health checkup, Vitamin D deficiency

## Abstract

**Purpose:**

We investigated and compared the serum 25-OH vitamin D [25(OH)D] level and prevalence of vitamin D deficiency (VDD) between patients who underwent total knee arthroplasty (TKA) and healthy participants through a matched analysis.

**Methods:**

The unmatched case group consisted of 824 patients who underwent TKA and the unmatched control group 2,794 healthy participants examined at our institution. The control group was matched on the various characteristics—sex, age, weight, body mass index (BMI), blood chemistry, and season of sampling—through propensity score matching (PSM). After PSM, 501 and 721 patients were matched in the case and control group, respectively. Levels of blood chemistry including 25(OH)D were examined and VDD was defined as < 20 ng/mL.

**Results:**

The average serum 25(OH)D level was significantly lower in the OA group (15.3 ng/mL) than that in the control group (19.9 ng/mL, p < 0.001). When categorized using a 20 ng/mL cutoff, the VDD prevalence was 75.0% in the OA group and 59.4% in the control group. The prevalence of vitamin D insufficiency was 18.4% in the OA group and 24.5% in the control group. The prevalence of vitamin D sufficiency was 6.8% in the OA group and 15.9% in the control group (p < 0.001).

**Conclusions:**

The patients who underwent TKA had lower serum 25(OH)D level and higher VDD prevalence compared to the healthy participants who matched using PSM. There were no differences in VDD rates by sex or obesity and the VDD prevalence was more than 70% during all season. Therefore, in patients undergoing TKA, general attention to VDD is required regardless of sex, obesity, and season.

***Summary*:**

Serum 25-OH vitamin D [25(OH)D] level and vitamin D deficiency (VDD) prevalence were compared between patients undergoing total knee arthroplasty and healthy individuals. The differences in serum 25(OH)D level and VDD prevalence were significant between the two groups after propensity score matching

**Supplementary Information:**

The online version contains supplementary material available at 10.1007/s11657-022-01097-7.

## Introduction

Vitamin D plays significant roles in the musculoskeletal system, such as on cartilage health, muscle strength, and fall prevention in the elderly [1–3]. Vitamin D status associated with the postoperative outcomes of patients undergoing various orthopedic surgeries [4–6], and patients with vitamin D deficiency (VDD) undergoing total knee arthroplasty (TKA) have significantly worse outcomes [7–9].

High rates of VDD have been demonstrated in various patient populations, including patients undergoing total joint arthroplasties [10]. Maier et al. [11] have reported that over 60% of patients had VDD in a study of 1,083 patients aged > 70 years and admitted to an orthopedic surgery department. Goula et al. [12] have evaluated the serum 25-OH vitamin D [25(OH) D] status in patients with knee or hip osteoarthritis (OA) scheduled for joint replacement and have reported that 81.7% of patients had VDD. Regarding the role of 25(OH)D level in TKA, Jansen and Haddad [8] have reported a high prevalence of VDD in elderly patients with advanced knee OA scheduled for TKA, and Piuzzi et al. [10] have reported that 26.9% of patients had VDD.

In a study using the National Health and Nutrition Examination Survey (NHANES) data collected from 2001 to 2004, the mean serum 25(OH)D level was 24 ng/mL and the prevalence of VDD was 77% [13]. In a study using the fourth Korea NHANES conducted in 2008, VDD was found in 47.3% of men and 64.5% of women [14].

To the best of our knowledge, no study has yet examined serum 25(OH)D levels and compared the prevalence of VDD in patients undergoing TKA and matched healthy individuals. Therefore, we performed a matched analysis of patients who underwent TKA and healthy individuals who were examined and compared the serum 25(OH)D level and VDD in the two groups. We hypothesized that there would be a significant difference in serum 25(OH)D levels or in the prevalence of VDD between the two groups in matched analysis.

## Methods

This study was approved by our institutional review board (UUH-2021–05-019), and informed consent was obtained from all study participants.

### Study design and setting

This was as a retrospective single-center study with a matched case–control design to address the purpose of this study. All study participants lived in a city located at 35.6 degrees latitude and were of a single Asian ethnicity.

### Patients

We performed a retrospective review of the medical records from January 2012 to December 2020. Based on the records, 898 patients underwent primary TKA. The patients recruited underwent TKA following presentation with moderate or advanced OA (Kellgren-Lawrence [15] grade III-IV, K-L grade III-IV) of the knee. First, patients with metabolic diseases, such as hyperparathyroidism and Paget’s disease, were excluded. Second, patients with serum 25(OH)D levels < 3 mg/mL and > 70 mg/mL were excluded. Then, the late laboratory findings of patients who underwent staged bilateral surgery were excluded because the serum 25(OH)D levels could be changed after the first surgery among staged bilateral TKA. Therefore, 824 patients were included in the case group (OA group). Meanwhile, the criteria for inclusion in the control group were at least 55 years of age, no history of TKA, and had undergone regular health checkup in our institution. Altogether, 2,794 participants were included in the control group.

### Propensity score matching strategy

The basic principle of propensity score matching (PSM) analysis was to estimate the propensity score (PS) for all observations in the case group and to match it with the observations in the control group with the closest PS value. Moreover, selection bias in selecting case and control groups can be reduced through PSM analysis, and any effect can be compared more accurately by controlling the influence of variables that can affect the results. The purpose of PSM analysis was to balance the two groups, not the number of observations in the two groups. Therefore, if there was no observation with the closest PS value in the control group, matching would not be possible. The reason why the number of observations was lost in the case group was the same with the reason as above. To reduce the bias and potential confounding among patients in the two groups, we performed careful adjustment for different distributions of baseline characteristics using a 2:1 PSM analysis with nearest-neighbor method based on a greedy matching algorithm that sorts by estimated PS. PSM was conducted by a 2:1 nearest-neighbor matching using a caliper size of 0.2. The balance of covariates in the matched groups was evaluated by measuring their standardized differences in means. All standardized mean differences in the baseline variables were < 0.20 (20%). We matched the groups according to sex, age, weight, body mass index (BMI), blood chemistry, and season of sampling. The blood chemistry included measurements of serum levels of alkaline phosphatase, calcium, and phosphorus, which are known to be associated with vitamin D level. The blood chemistry was a routine test item to evaluate a patient’s health condition before TKA; hence, it was planned to be used as a PSM analysis item. The program performs all analyses using the R software (version 4.0.2).

### Categorization of vitamin D level

The serum 25(OH)D levels were measured in routine blood samples taken from patients at their pre-admission visit using radioimmunoassay and checked for vitamin D status. The patients were categorized into three groups according to their vitamin D status. VDD, insufficiency, and sufficiency were defined as a serum 25(OH)D level of < 20 ng/mL, 20–29 ng/mL, and 30–150 ng/mL, respectively [16]. The distribution of vitamin D categories was checked in the two groups, and the vitamin D levels were compared by category in both groups.

### Obesity

BMI is a person’s weight in kilograms divided by the square of height in meters. If the patient’s BMI was ≥ 30.0, we classified the patient as within the obesity range [17].

### Season of sampling

The season of sampling was divided into spring (March, April, and May), summer (June, July, and August), autumn (September, October, and November), and winter (December, January, and February).

### Statistical analysis

Continuous variables are presented as mean with ranges. The Student’s t-test was performed to compare variables in unmatched patients and paired t-test was performed in matched patients. The Chi-square test was performed to verify the difference in the ratio of vitamin D categories between both groups. The regression analysis was performed to adjust the age between both groups. In the PS matched pairs, the risks of each outcome were compared by logistic regression using generalized estimating equations for categorical variables that accounted for the clustering of matched pairs. A p-value of < 0.05 was considered significant. Statistical analyses were performed using the SPSS version 24 (SPSS Inc., Chicago, IL, USA).

## Results

### Demographics

**Fig. 1:**
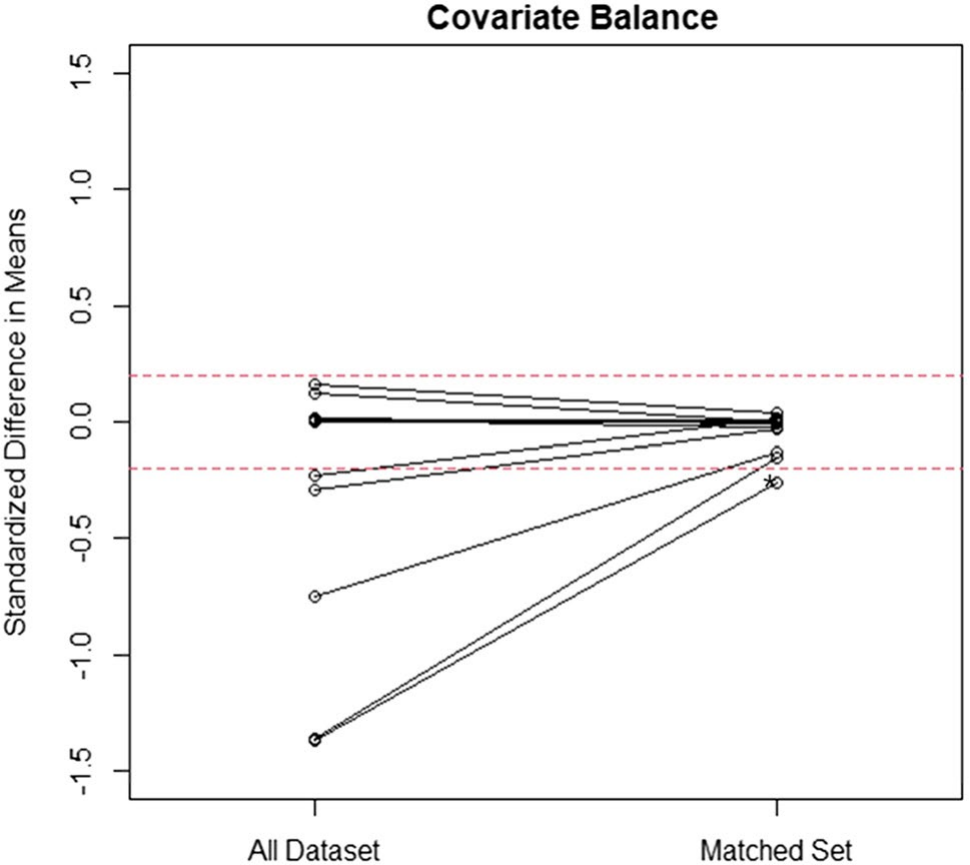
The balance of covariates in the all dataset and matched set was evaluated by measuring standardized differences in means. All standardized mean differences in the baseline variables were < 0.20, except for age (asterisk)

Before matching, the demographics of the two groups showed significant differences in age, sex, BMI, alkaline phosphatase, and phosphorus (Supplementary). The average age of the 3,618 patients was 63.7 (range, 55–90) years; 1,908 (52.7%) were women and 1,710 (47.3%) were men. The average weight was 63.5 (range, 32.3–103.4) kg, and the average BMI was 24.6 (range, 14.5–45.3) kg/m^2^. Here, a total of 1,222 patients were matched 2:1 through PSM, and no significant difference was found in all variables between the two groups, except for age (Fig. 1). The average age of the 1,222 patients was 67.3 (range, 55–90) years; 939 (76.8%) patients were women and 283 (23.1%) were men. The average weight was 61.6 (range, 34.8–102.0) kg and the average BMI was 25.3 (range, 14.9–40.2) kg/m^2^.

### Vitamin D status

**Fig. 2:**
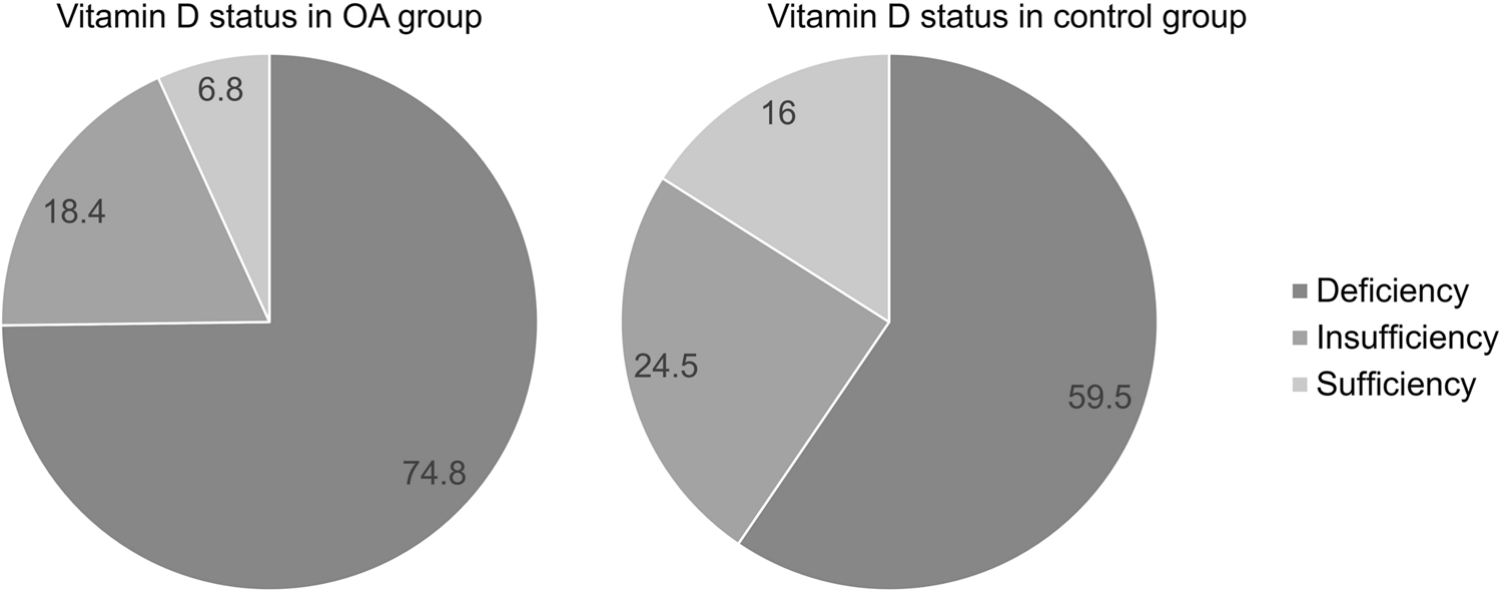
Vitamin D status in both group (OA and control group). The prevalence of vitamin D deficiency, insufficiency and sufficiency in the OA group was 74.8%, 18.4%, and 6.8%, respectively. The prevalence of vitamin D deficiency, insufficiency, and sufficiency in the control group was 59.5%, 24.5%, and 16.0%, respectively

The serum 25(OH)D levels of the patients were normally distributed, with a mean value of 19.1 (range, 3–66.6) ng/mL. The average serum 25(OH)D level was significantly lower in the OA group (15.3 ng/mL) than that in the control group (19.9 ng/mL, p < 0.001). When categorized using a 20 ng/mL cutoff, the VDD prevalence was 75.0% in the OA group and 59.4% in the control group (Fig. 2). The prevalence of vitamin D insufficiency was 18.4% in the OA group and 24.5% in the control group. The prevalence of vitamin D sufficiency was 6.8% in the OA group and 15.9% in the control group (p < 0.001). It was confirmed that average serum 25(OH)D level in the OA group was lower than the control group even after age was adjusted (OR 0.949; 95% CI 0.936–0.962; p-value < 0.001).

### VDD according to sex

**Fig. 3:**
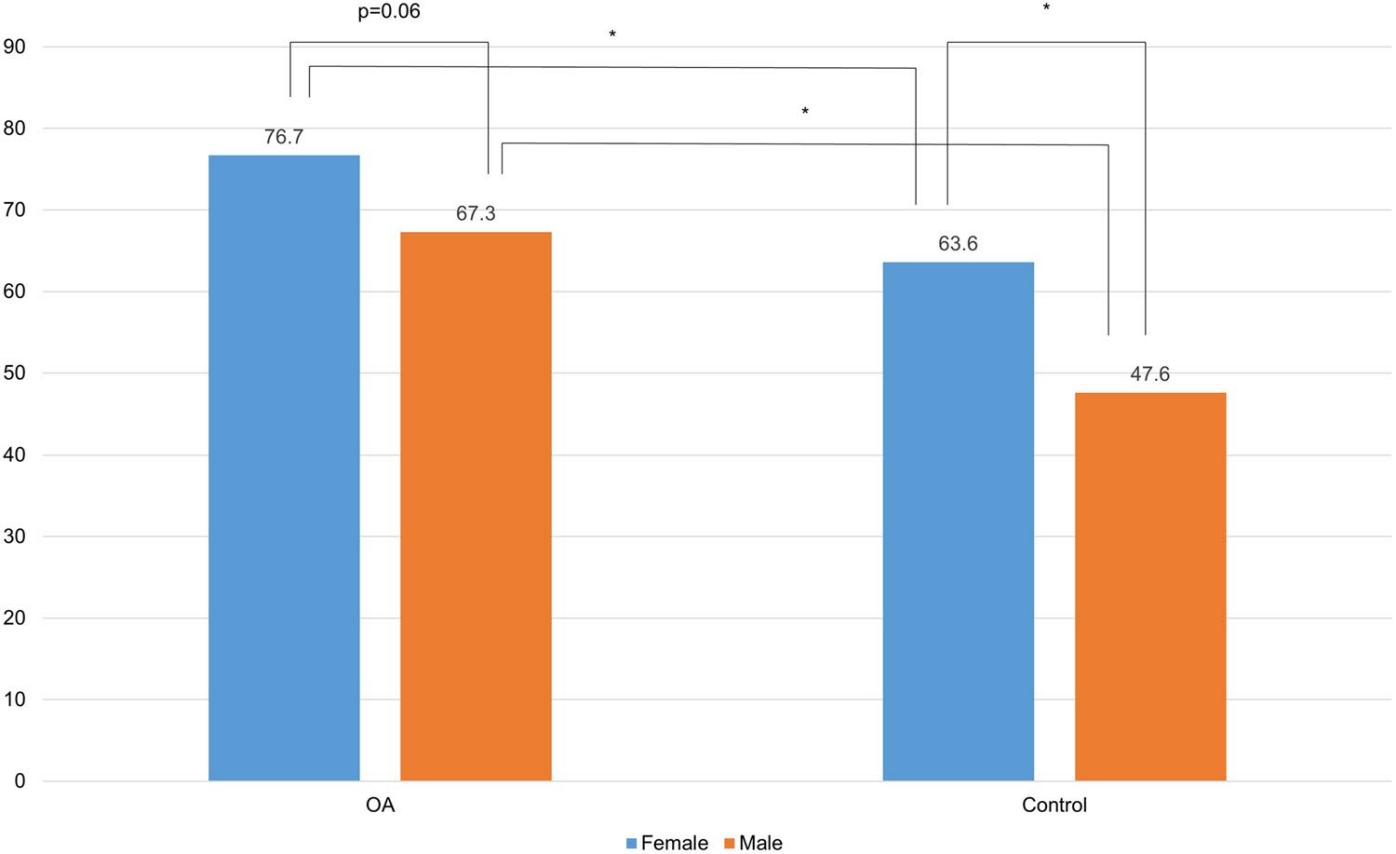
The percentage of vitamin D deficiency (VDD) according to sex in osteoarthritis (OA) and control group. The percentage of VDD in the OA group was significantly higher than in the control group for both sexes. There was no significant difference between female and male in the OA group (p = 0.06), and in the control group, the percentage of VDD in females (63.6%) was significantly higher than that of the males (47.6%). Asterisk (*) indicates p-value < 0.01

The VDD percentage according to sex is shown in Fig. 3. As a result of comparison between the same sexes, the percentage of VDD in the OA group was significantly higher than that in the control group for both sexes (female, p < 0.01; male, p < 0.01). As a result of comparing men and women in the same group, there was no significant difference between men and women in the OA group (p = 0.06), and in the control group, the percentage of VDD in women (63.6%) was significantly higher than that of men (47.6%).

### VDD according to obesity

**Fig. 4:**
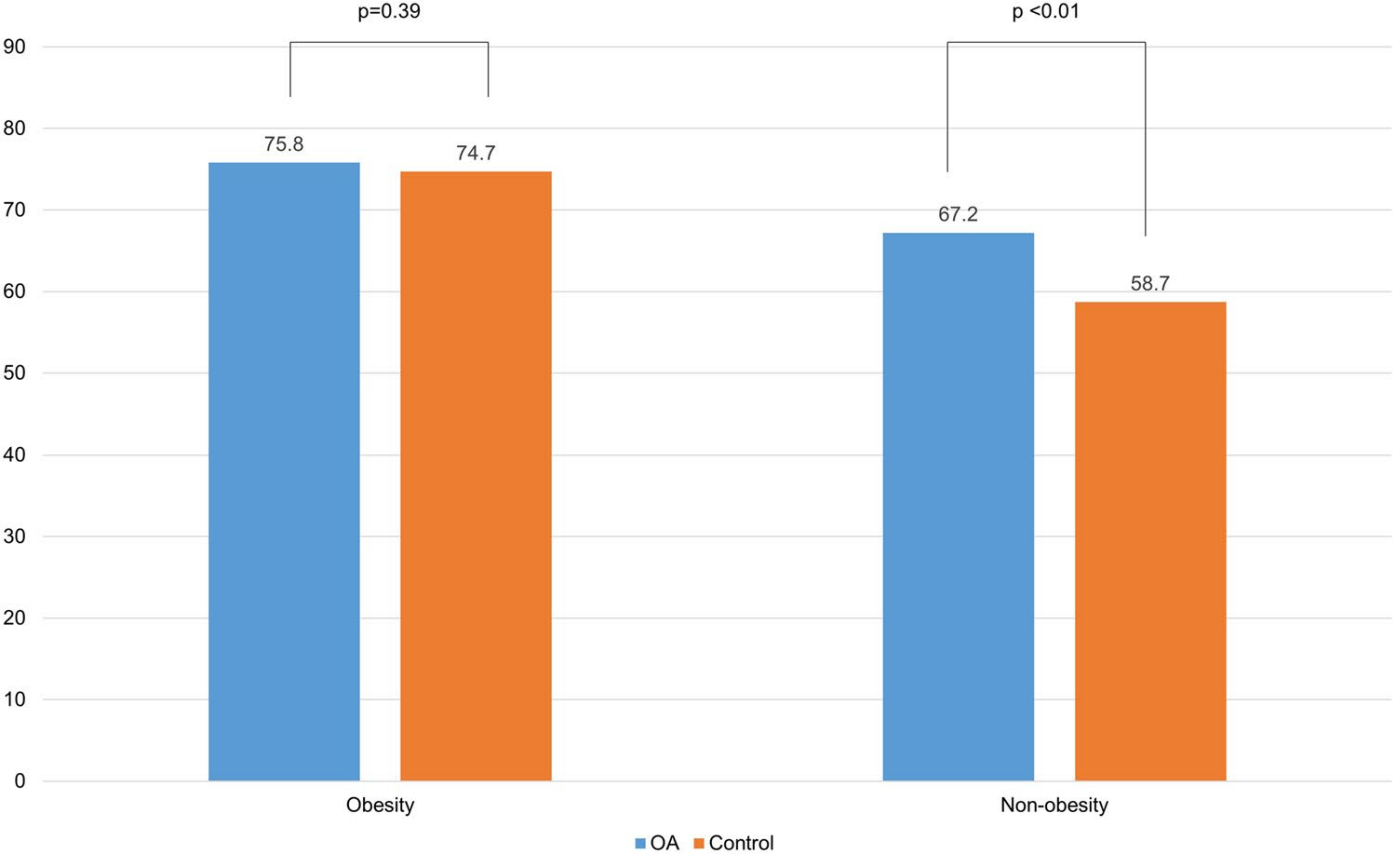
The percentage of vitamin D deficiency (VDD) according to obesity in osteoarthritis (OA) and control group. There was no significant difference in the percentage of VDD in the OA and control groups in obesity patients. In non-obesity patients, there was significant difference between the two groups

The VDD percentage according to patients’ obesity status is shown in Fig. 4. No significant difference was noted in the percentage of VDD in the OA group (75.8%) and control group (67.2%) in obesity patients (p = 0.39). However, in non-obesity patients, 74.7% (349/467) of the OA group had VDD, whereas 58.7% (387/659) of the control group had VDD; hence, significant difference was determined between the two groups (p < 0.01).

### VDD according to season of sampling

**Fig. 5:**
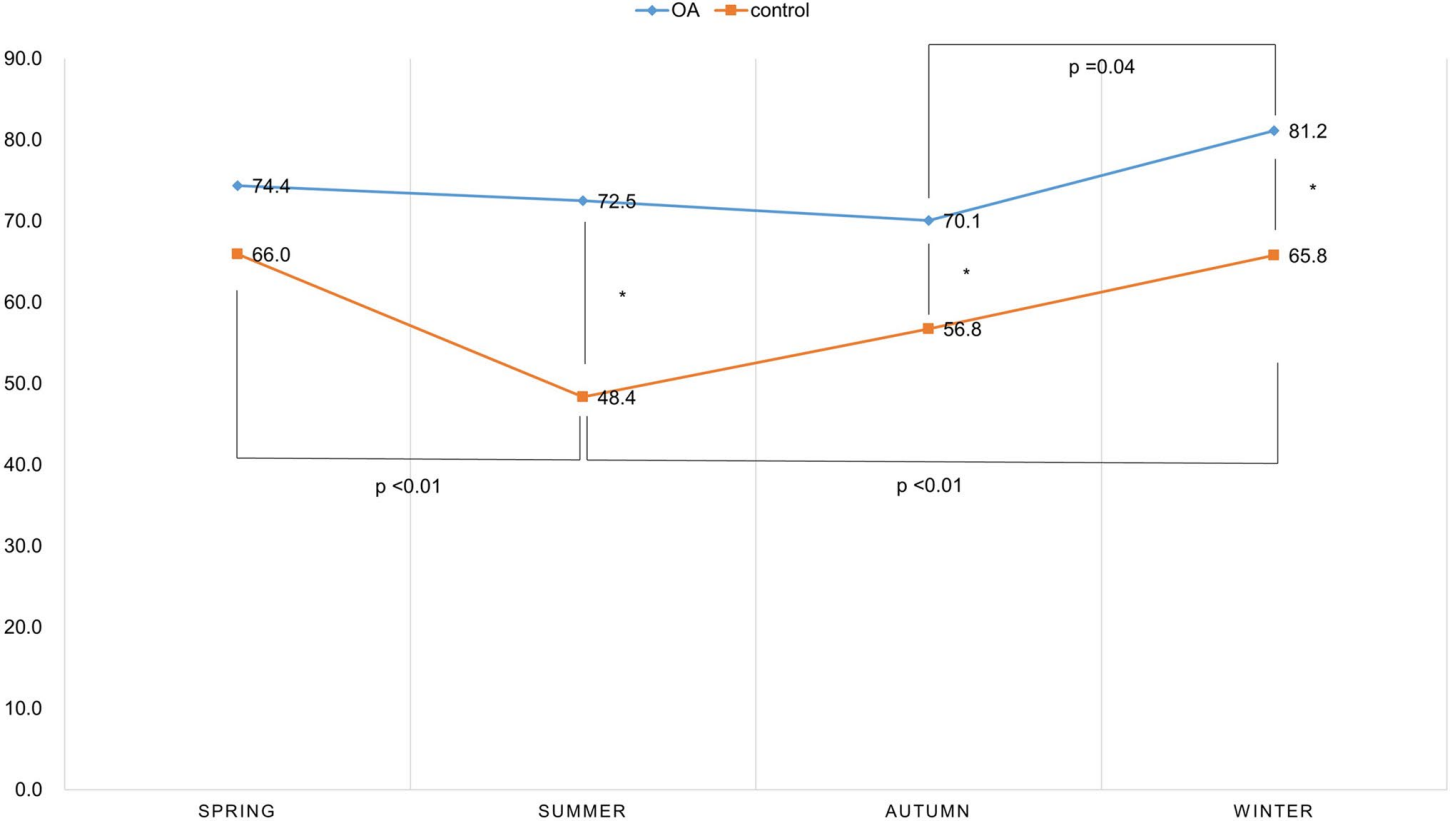
The percentage of vitamin D deficiency according to the season of sampling in the osteoarthritis (OA) and control group. Between the two groups, a significant difference was observed in the percentage of VDD in summer, autumn, and winter (asterisk). Seasonal differences in VDD prevalence in the OA group were significant only in winter and autumn. In the control group, there were significant differences in VDD prevalence between summer and spring and between winter and summer

Table 1 shows the percentage of vitamin D deficiency, insufficiency, and sufficiency by season of sampling. Between the two groups, there was a significant difference in the prevalence of VDD according to the seasons in summer, autumn, and winter, and the difference was the largest in summer (24.1% points, p < 0.001), followed by winter (15.4% points, p < 0.001), and autumn (13.3% points, p = 0.03) (Fig. 5). Seasonal differences in VDD prevalence in the OA group were significant only in winter (81.2%) and autumn (70.1%) (p = 0.04). In the control group, there were significant differences between summer and spring (p < 0.001) and winter and summer (p < 0.001).

**Table 1:** Vitamin D status according to season of sampling in osteoarthritis (OA) and control group

Group	Season of sampling	Vitamin D deficiency	Vitamin D insufficiency	Vitamin D sufficiency	Total
		n (%)			
OA	Spring	93 (74.4)	22 (17.6)	10 (8.0)	125
	Summer	95 (72.5)	26 (19.8)	10 (7.6)	131
	Autumn	75 (70.1)	23 (21.5)	9 (8.4)	107
	Winter	112 (81.2)	21 (15.2)	5 (3.6)	138
Control	Spring	124 (66.6)	40 (21.3)	24 (12.8)	188
	Summer	88 (48.4)	56 (30.8)	38 (20.9)	182
	Autumn	88 (56.8)	31 (26.5)	26 (16.8)	155
	Winter	129 (65.8)	40 (20.4)	27 (13.8)	196

## Discussion

This study had the largest cohort of patients undergoing TKA who were screened for VDD and compared with a matched healthy population. A high VDD prevalence was evident in patients who underwent TKA, with 75.0% of the OA group having 25(OH)D levels of < 20 ng/dL. Moreover, a significant difference was observed between the two groups.

Heidari et al. [18] determined the status of serum 25(OH)D levels in patients with knee OA compared with controls and have reported that in the entire population, the mean serum 25(OH)D level in patients with OA was not significantly lower than that of the controls. However, this study showed that the serum 25(OH)D level of the case group who had knee OA and underwent TKA was lower than that of the control group, and in that the average age of the total population in this study was 67.3 years, this study showed a different result from the previous study that the serum 25(OH)D level was low only in patients < 60 years of age among knee OA patients [18]. Bhattoa et al. [19] characterized the prevalence of VDD and vitamin D insufficiency in healthy men aged > 50 years and have reported that the prevalence of hypovitaminosis D was 52.9%. As a result of analyzing only men > 55 years of age in the control group of this study, the prevalence of hypovitaminosis D among 185 men with an average age of 68.4 was 75.7% (VDD, 47.6%; vitamin D insufficiency, 28.1%), which was higher than that of the previous study.

Arima et al. [20] have reviewed several epidemiological studies, which have assessed the correlation between serum 25(OH)D levels and OA in elderly individuals and have reported that data on the association between serum 25(OH)D levels and OA are contrasting. Although this study did not directly compare the OA grades of the two groups, TKA was generally performed in patients with a moderate or advanced grade of knee OA, and even with the same OA grade, the patients who had more severe pain underwent TKA. Therefore, this study’s results can be interpreted as the serum 25(OH)D level having an inverse association with the grade of knee OA, or the severity of pain caused by knee OA. The authors speculated that the reason for the decrease in serum 25(OH)D level in knee OA patients was that the overall number of activities including outdoor activities decreased due to knee pain and this is consistent with a previous study [21] that has evaluated the association between serum 25(OH)D level and physical activity. Klingberg et al. [21] explored parameters associated with 25(OH)D in a healthy adult population and serum 25(OH)D was significantly higher in the physically active population who performed physical exercise regularly at least once a week.

Goula et al. [12] have reported a significant positive association between serum 25(OH)D levels and the male sex in patients who were scheduled for knee or hip joint replacement. The result of a previous study can be interpreted as the reason that women have less outdoor activities than men [20]. However, in the present study, no significant difference was noted between men and women in the OA group (p = 0.06). The authors believed that the reason of the significantly higher VDD percentage for both men and women in the OA group compared to the matched control group was that patients in the OA group had severe knee pain enough to undergo TKA, and it is possible that the knee pain affected the outdoor activity level or sun exposure. Similarly, the authors believed that the reason why there was no significant difference in VDD percentage between men and women in the OA group was that patients with moderate or advanced OA had similar degrees of restriction of movement and decrease in outdoor activities due to similar severe knee pain.

Maier et al. [11] assessed risk factors for insufficient 25(OH)D levels in patients aged > 70 years and admitted to an orthopedic surgery department and have reported that patients presenting with obesity were more likely to have low 25(OH)D levels. In this study, after matching between the patients in the OA group and the control group, the standardized mean difference of BMI between the two values was < 0.2; however, the serum 25(OH)D level in the OA group was significantly lower than that of the control group. A significant difference was observed in BMI values by vitamin D status in the total population and control groups (p < 0.01, 0.011); however, in the OA group, the difference in BMI values by vitamin D status was not significant (p = 0.63). In the analysis of non-obesity patients, the VDD percentage of the OA group (74.7%) was significantly higher than that of the control group (58.7%). Regarding these results, the authors hypothesized that in the OA group, not only the association between serum 25(OH)D level and obesity but also a decrease in outdoor activity level and in sun exposure due to knee OA were added.

In general, VDD is known to decrease in summer [21], and in this study, it was confirmed that healthy participants in the control group significantly decreased VDD in summer compared to spring and winter. However, VDD was confirmed in more than 70% of OA patients who underwent TKA regardless of the season, and in winter, > 80% of patients had VDD. Regarding these results, the authors thought that it was because the outdoor activity level or sun exposure did not change according to the season in patients with severe knee pain enough to undergo TKA. The authors thought that the greatest difference (24% points) between the two groups appeared in summer because of the characteristics of the OA group, where VDD was high regardless of the season, and the general population, where VDD decreased in summer.

The strength of this study was that matched healthy participants were used to compare serum 25(OH)D level in patients who underwent TKA. However, this study had several limitations. First, we did not analyze whether the knee X-rays were taken or OA on X-rays was graded in the control group. However, for the reasons mentioned above, it can be estimated that the healthy participants in the control group had an OA grade less than K-L grade II or were not painful enough to receive TKA even if their OA grade on X-ray was higher than K-L grade III. Second, markers of bone metabolism, including serum parathyroid hormone were not analyzed. However, no difference in serum calcium and phosphorus levels between groups may represent similar calcium homeostasis in both groups. Third, 40% of patients in the case group were excluded after PSM. However, this was excluded during the process of matching the two groups. After matching, no demographic difference was noted between the two groups, and even after matching, a large population was still analyzed. Fourth, clinical outcomes could not be evaluated in the case group because this study compared case group that underwent TKA with a healthy control group. Fifth, a more severe knee OA had a lower bone mineral density (BMD) and serum 25(OH)D level [22]; hence, it is also a limitation that BMD was not used as a matching variable in this study. Sixth, although main analysis comparing the outcomes in the overall PS-matched groups is robust in terms of observed confounder balance, this may not be the case in the sub-group analyses given the matching was not repeated for each sub-group. Therefore, when readers interpret the results of the sub-group analyses, it should be noted that these sub-group analyses are secondary analyses and there is no way of telling the role of confounding in the sub-group comparisons due to matching being broken. Finally, because this study retrospectively analyzed data that was collected and organized only for specific items selected in advance, the levels of outdoor physical activity or sun exposure, well known as significant determinants of vitamin D status, could not be collected.

Nevertheless, this study showed that the serum 25(OH)D levels in the OA group was 15.3 ng/mL, which was significantly lower than that in the control group, and the prevalence of VDD in the OA group was 75.0%, which was significantly higher than that in the control group (59.4%). In the OA group, no differences were observed between males and females and according to obesity status. Additionally, patients in the OA group had more severe VDD in summer than healthy participants, and VDD was confirmed in more than 80% of patients in the OA group in winter.

## Supplementary Information

Below is the link to the electronic supplementary material.

Supplementary file1 (DOCX 17 kb)

## Data Availability

The raw data used in the current study are restricted to protect participant privacy.
