# Supplementary material for: The Prevalence of Vitamin D Deficiency in Patients Undergoing Total Knee Arthroplasty: A Propensity Score Matching Analysis
Source: Arch Osteoporos. 2022 Mar 23;17(1):53. doi: 10.1007/s11657-022-01097-7 (PMC8942953; doi:10.1007/s11657-022-01097-7)
Supplement: Supplementary file 1 — Supplementary file1 (DOCX 17 kb) [file 11657_2022_1097_MOESM1_ESM.docx]

**Supplementary.** Results of balance test

|  | | Baseline characteristics of all patients | | | | Baseline characteristics of propensity score matched patients | | | |
| --- | --- | --- | --- | --- | --- | --- | --- | --- | --- |
|  |  | OA group  (n=824) | Control group  (n=2,794) | SMD | p-value | OA group  (n=501) | Control group  (n=721) | SMD | p-value |
|  |  | n (%) | n (%) |  |  | n (%) | n (%) |  |  |
| Sex | Female | 721 (87.5) | 1,187 (42.5) | -1.3612 | 0.000 | 403 (80.4) | 98 (19.6) | -0.1537 | 0.013 |
|  | Male | 103 (12.5) | 1,607 (57.5) |  |  | 98 (19.6) | 185 (25.7) |  |  |
| Season | Spring | 218 (26.5) | 715 (25.6) | 0.0122 | 0.966 | 126 (25.1) | 188 (26.1) | -0.0097 | 0.868 |
|  | Summer | 198 (24.0) | 683 (24.4) |  |  | 131 (26.1) | 182 (25.2) |  |  |
|  | Autumn | 191 (23.2) | 658 (23.6) |  |  | 107 (21.4) | 155 (21.5) |  |  |
|  | Winter | 217 (26.3) | 738 (26.4) |  |  | 137 (27.3) | 196 (27.2) |  |  |
|  | | Mean±SD | Mean±SD | SMD | p-value | Mean±SD | Mean±SD | SMD | p-value |
| Age | | 70.09±6.06 | 61.79±5.80 | -1.3701 | 0.000 | 68.14±5.54 | 66.69±6.54 | -0.2617 | 0.0000 |
| Weight | | 62.23±9.79 | 63.83±10.36 | 0.1587 | 0.000 | 61.37±9.35 | 61.77±10.42 | 0.0399 | 0.552 |
| BMI | | 26.59±3.47 | 23.98±2.88 | -0.7511 | 0.000 | 25.56±2.94 | 25.19±3.32 | -0.1307 | 0.028 |
| ALP | | 73.27±23.80 | 67.78±20.74 | -0.2297 | 0.000 | 71.53±22.84 | 71.90±25.56 | 0.0162 | 0.079 |
| Ca | | 9.26±0.54 | 9.32±0.36 | 0.1167 | 0.002 | 9.30±0.54 | 9.30±0.39 | -0.0029 | 0.955 |
| P | | 3.61±0.56 | 3.45±0.53 | -0.2944 | 0.000 | 3.61±0.583 | 3.59±0.51 | -0.0309 | 0.578 |

*OA*, osteoarthritis; *SMD*, standardized mean difference; *SD*, standard deviation; *BMI*, body mass index; *ALP*, alkaline phosphatase; *Ca*, calcium; *P*, phosphorus
